# Effects of a neonicotinoid pesticide on honey bee colonies: a response to the field study by Pilling et al. (2013)

**DOI:** 10.1186/s12302-015-0060-7

**Published:** 2015-11-06

**Authors:** Peter Paul Hoppe, Anton Safer, Vanessa Amaral-Rogers, Jean-Marc Bonmatin, Dave Goulson, Randolph Menzel, Boris Baer

**Affiliations:** 1 Am Hauenstein 13, 67157 Wachenheim, Germany; 2 Institute of Public Health, Ruprecht-Karls-University, INF324, 69120 Heidelberg, Germany; 3 Buglife-The Invertebrate Conservation Trust, Bug House Ham Lane, Orton Waterville, Peterborough, PE2 5UU UK; 4 CNRS Center for Molecular Biophysics CBM, Rue Charles Sadron, 45071 Orleans Cedex 2, France; 5 School of Life Sciences, University of Sussex, Falmer, Brighton, BN1 9QG UK; 6 Institute for Biology, Neurobiology, Free University of Berlin, Königin-Luise-Str. 28/30, 14195 Berlin, Germany; 7 Centre for Integrative Bee Research (CIBER), ARC Centre of Excellence in Plant Energy Biology, Bayliss Building, The University of Western Australia, Crawley, 6009 Australia

**Keywords:** Thiamethoxam, Honeybee, Field study, Critical review

## Abstract

Our assessment of the multi-year overwintering study by Pilling et al. (2013) revealed a number of major deficiencies regarding the study design, the protocol and the evaluation of results. Colonies were exposed for short periods per year to flowering oilseed rape and maize grown from thiamethoxam-coated seeds. Thiamethoxam as the sole active ingredient was used, not a more efficacious commercial product, at seed treatment rates that were lower than recommended as per common agricultural practices. Design and adherence to the protocol were described inadequately making it doubtful whether the study was implemented in a traceable way. No results are given for overwintering losses. Much emphasis is laid on presenting condensed raw data but no statistical evaluation is provided. Therefore, the work presented does not contribute new knowledge to our understanding of the potential impact of thiamethoxam products under field conditions. Furthermore, the authors express concern over the refereeing process of the paper. Publications in refereed journals are likely to be taken seriously in political debates and policy-making, and so must be based on truthful data and methodologies.

## Background

A 2-year moratorium implemented by the European Commission on the usage of several insecticidal neonicotinoids became effective on Dec 1, 2013. The manufacturers Bayer CropScience and Syngenta subsequently challenged the restrictions implemented juristically. Both companies argue that the bans’ justification is solely derived from evidence from laboratory studies showing adverse effects of neonicotinoids on individual honeybees whereas field studies with bee colonies demonstrate the absence of adverse effects. Similarly, a DEFRA paper [1], concluded “*that effects on bees do not occur under normal circumstances”,* and that *“laboratory based studies demonstrating sub*-*lethal effects on bees from neonicotinoids did not replicate realistic conditions, but extreme scenarios.”* Consequently, DEFRA supports the view that *“the risk to bee populations from neonicotinoids, as they are currently used, is low.*” To support this view, Pilling et al. [2] published the results of a field study that had been commissioned by the manufacturer of thiamethoxam (Syngenta) as part of the docket for thiamethoxam approval. In essence the study concludes that systemic residues in maize pollen and in nectar and pollen of oilseed rape (OSR) grown from thiamethoxam-treated seeds constitute a “*low risk*” for honeybee colonies [2].

We challenge the validity of this study and the conclusions drawn by providing a number of issues where the experimental approach, methodology, data reporting and analysis, and publication process are unclear, misleading or problematic. We conclude that the study is unable to provide any scientific insights into the effects of thiamethoxam on bee colonies in the field.

## Usage of a non-commercial product for experiments

The objective of the field study was to assess the “*specific effects of thiamethoxam”.* For this purpose, the active ingredient only was used for seed dressing and not a commercial product. Commercial products typically contain various co-formulants to protect the active ingredient from degradation, to promote uptake by the plant (e.g. surfactants, emulsifiers) and to increase effectiveness (for example by adding piperonyl butoxide). Hence, commercial products have higher biological activity and toxicity than the active ingredient alone [3]. Furthermore, commercial neonicotinoid products can also contain fungicides which further increase toxicity and efficacy of the active ingredient. For example, the acute toxicity (LD_50_, 24 h) of the neonicotinoid thiacloprid increased a 1000-fold when co-administered with a DMI fungicide [4]. For these reasons, the European and Mediterranean Plant Protection Organization (EPPO) recommended the usage of commercial products when conducting field trials [5]. Hence, the data obtained by Pilling using the active ingredient solely do not reflect a commercial situation in the field and consequently do not allow general conclusions about the impact of thiamethoxam products on bee health in the field.

## Pesticide concentrations for seed treatment were lower than declared

Maize and OSR seed were dressed with thiamethoxam at “*the maximum approved label rate*”, using 12.6 g a.s./ha for oilseed rape and 88.2 g a.s./ha for maize. However, according to the European Food Safety Authority (EFSA) [6], the maximum approved rate for OSR is 42 g a.s./ha; hence, the authors only used 30 % of the maximum approved rate. Similarly, the maximum approved rate for maize is actually 101 g a.s./ha, so the authors have used 87 % of the maximally allowed dosage. No explanation is given for the deviation of actual values from the target values.

## Flaws in the experimental design

(a) Colonies of treatment and control were separated by “*about 2* *km*″. However, this distance is inadequate to prevent cross-foraging of control bees in treated fields or vice versa, as honeybees have known limit of foraging ranges between 6 and 10 km [7]. Furthermore, according to the authors, each site was “*isolated*” from other bee-attractive crops and other maize and rapeseed fields, but no evidence is provided how such “*isolation*” was practically achieved in the field, and this would be very difficult to do in most arable farmland areas. Conversely, if the experimental plots were planted in areas unsuitable for arable farming, then one might expect that flowering of the crop would be poor. We would normally expect the locations of studies to be provided, for example by aerial photography or GPS coordinates.

(b) The time window of exposure in maize ranged from 5 to 8 days in 2006–2008 and from 19 to 23 days in 2009. In OSR, it ranged from 11 to 22 days. Such short exposure times are not field-relevant. Managed bee hives would be exposed to contamination via a variety of crops, dust, soil, water and weedy plants for significantly longer periods than just 23 days maximum.

(c) For most of the year (up to 360 days) the colonies were maintained in woodland sites “*without extensive agricultural crops*”. This does not reflect normal beekeeping practice in which bees are likely to be exposed to pesticides for much of the year. Because the exact locations are not disclosed as latitudes and longitudes, which is typically provided for ecological studies of this type, it is not possible to verify that colonies were indeed kept distant enough from other pesticide-treated agricultural crops. This is crucial given that exposure to other pesticide-treated crops is a normal situation for honeybees in agricultural landscapes, especially because of their substantial foraging ranges as mentioned above.

(d) The pesticide contamination history of the study fields was not assessed. Because agricultural soils are widely contaminated with a range of pesticides including long-lasting neonicotinoids such as imidacloprid, contamination history constitutes a possible confounder that may obscure detection of differences between treatment and control.

(e) During the field study, stored pollen was examined for thiamethoxam and clothianidin (the main metabolite of thiamethoxam) only, but not for other pesticides. Thus, the purported lack of difference between treated and control hives is arbitrarily attributed to the lack of effect of thiamethoxam rather than to the totality of confounding factors.

(f) Cumulative colony mortality from all causes was 42 colonies (70 %) of the total of 60 colonies initially set up to conduct the experiment (maize and OSR, treatment and control, across 4 years). The main cause given in Table 2 is “*lost due to male brood only*”. High prevalence of male brood has been linked to the impact of neonicotinoids [8]. These substantial losses resulted in a substantial increase of missing observations throughout the experiment, because sample sizes must have been bigger at the start of the experiment when no differences were expected compared to the end. The details of how colony losses were accounted for during the data analysis remained unclear, but would have required statistical procedures to provide measure of confidence for the claims that there were no significant treatment effects.

(g) Surprisingly, no data are reported for colony losses during winter, although this was a main objective of the study. Instead, the authors claim that control and treatment colonies had “*successfully overwintered*”. Upon our request, the authors provided us with raw data collected at “*the first assessment of the year*” which was mostly in March. According to these raw data, the total number of colonies found dead from all causes (OSR and maize, all sites, across all years) was 19. However, because further assessments were made in late March and also on several dates of April (see legends to Figures 9 and 10 [2]) the data provided do not reflect total winter losses. Upon including losses that occurred in March and April (Table 2 [2]), we arrive at cumulative total winter losses of 23 colonies (OSR and maize, all sites, all years). All but four of these are attributed to queen failure and the consequent presence of “*male brood only*”. As such colonies are unable to survive winter they should have been included in winter mortality as suggested earlier [9]. Notably, as colony results are reported “*as mean values of six hives”* it is unclear how lost colonies or missing observations were substituted for.

(h) In summary, the deficiencies in planning, executing, evaluating and reporting of this study become more obvious by comparison with a very recent field study [10].

## Lack of statistical analysis

The results are presented in 24 figures and one table. Data are presented as averages, but no measures of variance are provided and there is not a single statistical analysis. The authors conclude on page 9 that “*Whilst it would, in principle, be possible to carry out a formal statistical analysis for both maize and the oilseed rape data …. a formal statistical analysis was not conducted because this would be potentially misleading.*” This means that the entire paper and its conclusions rely on the readers’ visual inspection of graphs, and the authors conclude on page 9: “*… it is clear from simple inspection of the results that no large treatment effects were present*” [2]. In fact several of the Figures do not appear to support this statement. For example, the graphs presented for years 2006 and 2008 in Figure 6 imply substantially higher mean numbers of dead bees in the control hives compared to the treated hives. Another example is the data presented in Figure 11 for years 2007 and 2008, showing that maximum hive weight consistently differed over time between the treatments. However without providing measures of variance in these Figures, it remains unclear whether there was no difference between treatments. More importantly though, it is standard scientific practice that quantitative data require proper statistical analyses to support claims, and to determine whether or not two sets of data differ from each other. Consequently, with the complete absence of statistical testing, no conclusions about the effects of thiamethoxam on colony health can be drawn.

## Inadequate refereeing process

Given these issues about experimental design and data analysis, we were surprised that the paper fulfilled the publishing journal’s policy regarding experimental design and data analysis. When we checked the author’s guidelines for publication on the journals website, we found that “*Experiments, statistics, and other analyses are performed to a high technical standard and are described in sufficient detail”* [11]. We concluded that under these requirements, the Pilling study is unable to fulfill these criteria. Because the authors were clear about their experimental design and the lack of data analysis in their paper, we became even more puzzled to understand the reasons or arguments that led to the eventual acceptance of this manuscript.

However, as part of our investigations, we were able to identify some of the referees that handled the paper during the publication process, some of which expressed frustration about their refereeing experience for this paper. In addition, one referee had been working for a Government department in pesticide regulation. E-mail exchanges between this referee and the authors during the refereeing process show an exchange of information about the submitted manuscript and the ongoing review process. Subsequent to reviewing the paper, the referee became an employee of Syngenta and later appeared as an author of the paper at an international conference organized by the International Union for the Study of Social Insects (IUSSI) in Cairns 2014. We therefore concluded that the refereeing process failed to satisfy international standard scientific procedures, which could therefore explain the eventual acceptance and publication of the manuscript.

## Conclusions

We conclude that the 4-year field study by Pilling et al. [2] exposing honeybees to thiamethoxam-treated crops has substantial scientific deficiencies. These include limited exposure periods, the use of pure thiamethoxam instead of a commercial preparation, the failure to quantify colony losses in winter and the fact that 70 % of colonies did not survive to the end of the experiment, as well as the lack of any statistical evaluation and a compromised reviewing process. Hence, the data and analyses presented do not allow any of the conclusions drawn by the authors concerning risks of systemic residues of thiamethoxam on honeybee colonies.
